# Editorial: Defective macroautophagy in organelle turnover: From basic mechanisms to human disease

**DOI:** 10.3389/fcell.2022.1018778

**Published:** 2022-09-09

**Authors:** Eloy Bejarano, José Antonio Rodríguez-Navarro, Roberta Filograna, Javier Calvo-Garrido

**Affiliations:** ^1^ School of Health Sciences and Veterinary School, Universidad CEU Cardenal Herrera, CEU Universities, Valencia, Spain; ^2^ Servicio de Neurobiología, Departamento de Investigación, Hospital Ramón y Cajal, IRYCIS, Madrid, Spain; ^3^ Department of Cell Biology, Complutense University of Madrid, Madrid, Spain; ^4^ Department of Medical Biochemistry and Biophysics, Karolinska Institutet, Stockholm, Sweden

**Keywords:** macroautophagy, selective autophagy, neurodegeneration, aging, SQSTM1

Macroautophagy is a highly conserved intracellular degradation system in charge of intracellular cleaning. Double-membrane vesicles called autophagosomes sequester cytoplasmic content for degradation in different organisms, from yeast to mammals. Although initially macroautophagy was described as a cytoplasmic bulk degradation process triggered by the lack of nutrients to balance energy during starvation, an increasing literature support that macroautophagy contribute to the proper organelle turnover in a selective manner. Within the last years different autophagic receptors have been identified and characterized some of their functions.

**FIGURE 1:**
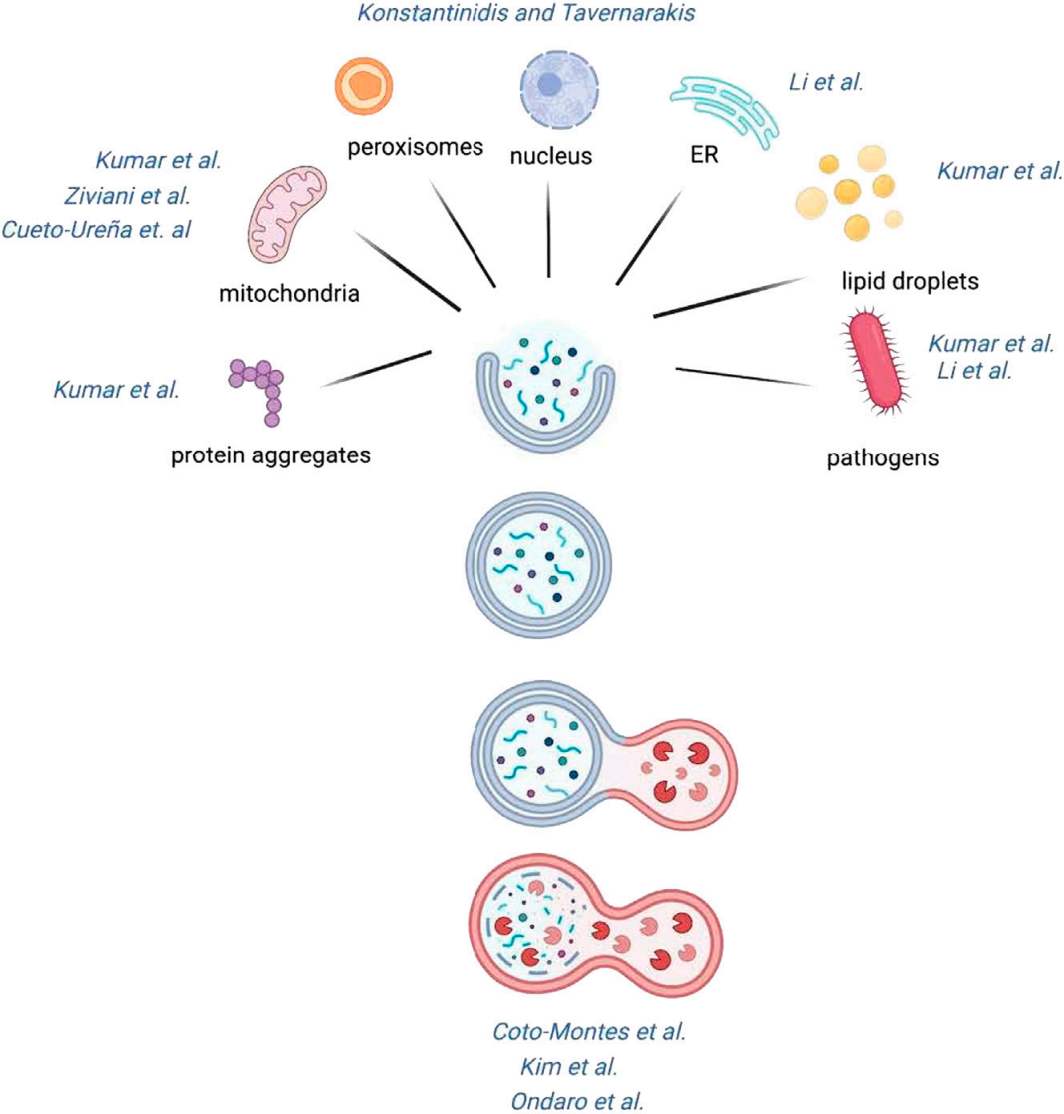
Schematic representation of selective macroautophagy. Specific cargos, such as dysfunctional organelles or invading microbe, are initially sequestered by the phagophore and engulfed in the forming double-membrane autophagosome. The autophagosome’s outer membrane subsequently fuses with the lysosome releasing the enclosed material in the autolysosome, where the sequestered cargos are degraded by hydrolases.

Organelle-specific autophagy remove damaged or obsolete organelle instead of random portions of cytoplasm to preserve organelle function. Multiples organelles including mitochondria (mitophagy), peroxisomes (pexophagy), endoplasmic reticulum (ER-phagy), lipid droplets (lipophagy) and other organelles are engulfed by autophagosomes and targeted to the lysosomes for degradation. Also, selective autophagy in immune cells plays a major role in the capture of pathogens (xenophagy) (Figure 1). An increasing literature show that different autophagic receptors and adaptors work together in the selection phase so autophagosomes can enwrap specific cargo and a defective performance of these autophagy proteins is associated to multiple pathologies and age-related disorders.

In this Research Topic we focus on molecular insight into the modulation of organelle turnover as well as how the deregulation of this process can lead to pathophysiological conditions. Our knowledge about functional aspects of these receptors and adaptors receptors has exponentially increased throughout the past few years. This in-depth understanding at molecular level could provide therapeutic tools to assist a more efficient design of strategies to combat diseases in which autophagy is defective. This Research Topic includes seven review articles and one original research article that highlight recent advances in the field of defective macroautophagy in organelle turnover and disease.

In the review article “*Selective Autophagy Receptor p62/SQSTM1, a Pivotal Player in Stress and Aging*”, Kumar et al. summarize the current knowledge on the protein Sequestosome 1 (p62/SQSTM1) a multifaceted protein involved in maintaining the cellular homeostasis. p62 is not only a selective autophagy receptor involved in clearance of aggregates or mitochondria but is also involved in ubiquitin-proteasome system, cellular metabolism, signaling, and apoptosis. This article discusses role of p62 in coordinating the ubiquitin-proteasome system and autophagy and highlight the implication of p62 function on aging and neurodegeneration.

Li et al. contribute to this Research Topic with the review article “*ER-Phagy and Microbial Infection*” where the authors give a comprehensive overview of the current understanding on the process of autophagic clearance of ER fragments termed ER-phagy or reticulophagy, a vital mechanism of quality control for this organelle. The authors summarize the function of ER-phagy as a host defense mechanism when pathogens infect cells and describe how deficiency of ER-phagy facilitates viral infection.

Konstantinidis and Tavernarakis also contribute to this Research Topic with a mini review article “*Autophagy of the Nucleus in Health and Disease*”. The authors highlight recent advances in nucleophagy, an organelle-selective subtype of autophagy that targets nuclear material for degradation, and describe nucleophagic events in the context of pathology, such as, cancer, DNA damage, ageing and neurodegeneration.

Also, focusing on the role of autophagy in neurodegeneration, Kim et al. contribute to this Research Topic with the review article “*Autophagy in the Neuronal Ceroid Lipofuscinoses (Batten Disease)*”, a family of neurodegenerative diseases featured by accumulation of autofluorescent lipoprotein aggregates, called ceroid lipofuscin, in neurons and other cell types outside the central nervous system. Mutations in *CLN* genes are associated to these neurodegenerative diseases and the authors summarize the current knowledge linking CLN mutations and altered autophagic pathways.

Also, Ondaro et al. contribute to this Research Topic with the review article “*Defects of Nutrient Signaling and Autophagy in Neurodegeneration*”. The authors provide a detailed overview of the pathways and processes involved in the mechanisms of nutrient sensing and autophagy under physiological conditions and highlight the autophagic organelle renewal essential to balance nutritional needs through the mobilization of internal energy stores. The authors describe how a defective autophagy-dependent balance of cellular energy participate in the molecular pathological events of different neurodegenerative diseases including Parkinson’s disease, frontotemporal dementia, amyotrophic lateral sclerosis, or Alzheimer’s disease.

Three more articles in this Research Topic focus on metabolic functions of selective autophagy in the context of mitochondria function and oxidative stress. In a mini-review presented by Coto-Montes et al. entitled “*The Interactome in the Evolution From Frailty to Sarcopenic Dependence*” a preponderant role of autophagy in interactome control is highlighted. The authors discuss the relationship between overweight and autophagic capacity in the muscle of the elderly.

In the original research article “*Rnd3 Expression is Necessary to Maintain Mitochondrial Homeostasis but Dispensable for Autophagy*”, Cueto-Ureña et al. report on the role of Rnd3 in mitochondrial homeostasis and autophagy. The authors find that Rnd3 a Rho GTPase protein necessary for the correct development of the central nervous system is not involved in autophagy and mitochondrial turnover. However, they conclude that the lack of Rnd3 causes an alteration of mitochondrial oxidative metabolism, mimicking the effect of oxidative stress.

Finally, Mauri et al. address the role of mitochondrial clearance in the sleeping brain. Extensive studies have been made using immortal human cells exogenously expressing Parkin followed by the treatment with mitochondrial oxidative phosphorylation uncouplers as CCCP. The authors point out to the lack of physiological relevance of the PINK1/Parkin pathway in mitophagy in *in vivo* models as mouse and flies. The authors focused on the role of the circadian clock in the regulation of mitophagy.

Altogether, this compilation delivers a focused picture of macroautophagy in organelle turnover and the impact of defective function of selective autophagy receptor in human disease and aging. However, other types of selective autophagy pathways such us lysophagy, ribophagy, glycophagy or pexophagy were not covered in this Research Topic. A vast research effort is currently focused on the identification of molecular determinants highly specific for each type of selective autophagy. These include receptors, interactors, posttranslational modifications as well as splicing variants. Alterations in these molecular determinants could be behind aging and aging-related diseases. Our knowledge about the topic is growing exponentially and will serve to help in the treatment of multiple human diseases.

